# Oncometabolites: A new insight for oncology

**DOI:** 10.1002/mgg3.873

**Published:** 2019-07-18

**Authors:** Fatemeh Khatami, Seyed Mohammad Kazem Aghamir, Seyed Mohammad Tavangar

**Affiliations:** ^1^ Urology Research Center Tehran University of Medical Sciences Tehran Iran; ^2^ Chronic Diseases Research Center, Endocrinology and Metabolism Population Sciences Institute Tehran University of Medical Sciences Tehran Iran; ^3^ Department of Pathology, Dr. Shariati Hospital Tehran University of Medical Sciences Tehran Iran

## Abstract

The new aspect of oncometabolite can be an indicator of genetic and epigenetic change and cancer biomarker.

Cancer is the uncontrolled cell behavior occurring as a result of several genetic and epigenetic alterations with the aim of the survival of tumors and metastasis. These mutations are directly linked to the metabolite change as the distinctive characteristic of several cancer types. Alterations in metabolite levels support tumor genesis in the course of various mechanisms like aerobic glycolysis, reduced oxidative phosphorylation, and the increased generation of biosynthetic intermediates (Yang, Soga, & Pollard, 2013). Finding the metabolic transformation apparatus can offer opportunities to recognize tumor tissue noninvasively, tumor behavior, or even stopping the tumor progression by blocking essential pathways (DeBerardinis & Chandel, 2016). Oncometabolite refers to metabolites whose great quantity were elevated noticeably in tumors compared with normal cells (Nowicki & Gottlieb, 2015). The first highlighted oncometabolite is D‐2‐hydroxyglutarate (D2HG) form of the TCA cycle intermediate α‐ketoglutarate which is in limited quantity in normal tissues but is at a higher concentrations in cancer cells (Xu et al., 2011). This oncometabolite is the result of isocitrate dehydrogenase 1 or 2 (IDH1 or IDH2) gene mutation commonly occurring in gliomas and acute myelogenousleukemias (AMLs) (Vatrinet et al., 2017; Yan et al., 2009). Increasing amount of D2HG can alter the function of dioxygenases requiring α‐ketoglutarate and epigenome of the cell like prolylhydroxylation of 5‐methyl cytosine in DNA and inhibition of histone lysine demethylases (Figueroa et al., 2010; Losman et al., 2013). Now oncometabolites can be evaluated as cancer biomarkers found in serum, plasma, urine, saliva, and tumor tissue samples (Beger, Schnackenberg, Holland, Li, & Dragan, 2006). There are nine famous oncometabolites including; 2‐hydroxyglutarate, glucose, fumarate, succinate, sarcosine, glutamine, asparagine, choline, and lactate suggested as biomarkers in glioma, prostate‐, gastrointestinal‐, breast‐, and endocrine‐related cancers (Khan et al., 2013; Yang, Soga, Pollard, & Adam, 2012). Although histological analysis and immunohistochemical studies have focused on primary tumor diagnosis or tumor recurrence, some new evidence of tumor markers in blood of cancer patients would provide more tumor diagnostic and prognostic evidence (Alimoghaddam et al., 2006; Haghpanah, Shooshtarizadeh, Heshmat, Larijani, & Tavangar, 2006; Khatami et al., 2017; Mohammadi‐asl et al., 2011; Tavangar et al., 2010). These circulation biomarkers can improve the integration of cell cycle progression, molecular tumorgenesis pathways, oncometabolism, and epigenetics to novel cancer diagnostic and therapeutic biomarkers (Khatami, Larijani, & Tavangar, 2018; Khatami & Tavangar, 2018).

In the near future, the noninvasive biomarkers like oncometabolites can help the oncologist to make decisions more precisely in shorter time, especially in predicting more aggressive tumor behavior (Dando et al., 2019). Over the last two years, huge number of research is published in which the role of oncometabolite is highlighted not only in cancer diagnosis but also in cancer management and prognosis (Ganapathy, 2018; Hanover, Chen, & Bond, 2018; Park et al., 2018). The applications of oncometabolomics as the therapeutic targets in drug discovery and precision medicine has been considered very recently (Galluzzi, Kepp, Vander Heiden, & Kroemer, 2013; Wishart, 2016). Many of the most‐effective medications are enzyme inhibitors, and thousands of metabolite‐inspired enzyme inhibitors or antimetabolites which have been already identified (Copeland, Harpel, & Tummino, 2007). The 2HG, suggested as an identifier oncometabolite of brain cancer and leukemia patients is a potential target of drug that targets their coding gene IDH1(Garber, 2010). Hhexokinase 2 is a major contributor to high glycolysis and lactate production, hence targeting hexokinase 2 in the rabbit VX2 tumor model for liver cancer was suggested ( Ko, Pedersen, & Geschwind, 2001). The small molecule 3‐bromopyruvate (3BP), a glycolysis inhibitor, has a potent anticancer activity and has the capacity to eradicate cancers in different animal models (Ko et al., 2004; Pedersen, 2012). Another gluco‐regulatory kinase is phosphofructokinase (PFK) correlated with the growth rate of Morris hepatomas transplanted in rats and also correlated with lactate production so inhibition of phosphofructokinase activity represented a logical target for inhibition of malignant tumor growth (Schaftingen, Jett, & Hue, 1981). Some amino acids like glutamine are of hexosamines, nucleotides, and nonessential amino acids essential for cell growth (DeBerardinis et al., 2007; Sarmadi, Izadi‐Mood, Sotoudeh, & Tavangar, 2009). Glutaminolysis in many cancers is the prominent target in cancer therapy and compounds such as 6‐diazo‐5‐oxo‐l‐norleucine (DON) and acivicin showed cytotoxic effects against several malignant tumors types like leukemia and colorectal cancers (Wise & Thompson, 2010). The number of malignant cancer inhibition through its oncometabolites and critical metabolic pathway are increasing.

Taking everything into the consideration, it is concluded that the cell cycle progression and cell survival are completely dependent on availability of the key metabolites. Oncometabolites of cancer cells directed them to certain metabolic pathways to sustain their uncontrolled growth through their genetic and epigenetic. They can be used both as biomarkers in cancer diagnosis panel and therapeutic targets in cancer management panels.
